# Cefiderocol: An Overview of Its *in-vitro* and *in-vivo* Activity and Underlying Resistant Mechanisms

**DOI:** 10.3389/fmed.2021.741940

**Published:** 2021-12-07

**Authors:** Jiahui Yao, Jin Wang, Mengli Chen, Yun Cai

**Affiliations:** ^1^Center of Medicine Clinical Research, Department of Pharmacy, Medical Supplies Center, People's Liberation Army of Chinese General Hospital, Beijing, China; ^2^Department of Pharmacy, Medical Supplies Center, People's Liberation Army of Chinese General Hospital, Beijing, China

**Keywords:** Gram-negative bacteria, activity, cefiderocol, resistance mechanisms, cephalosporins (therapeutic use)

## Abstract

Treatment of multidrug-resistant (MDR) Gram-negative bacteria (GNB) infections has led to a global public health challenging due to the bacterial resistance and limited choices of antibiotics. Cefiderocol (CFDC), a novel siderophore cephalosporin possessed unique drug delivery systems and stability to β-lactamases, has the potential to become first-line therapy for most aggressive MDR Gram-negative pathogens infection. However, there have been reports of drug resistance in the course of using CFDC. This study provides an overview of the *in-vitro* and *in-vivo* activity of CFDC and potential resistance mechanism was also summarized. In general, CFDC shows excellent activity against a broad range of MDR GNB pathogens including *Enterobacteriaceae, Klebsiella pneumoniae, Pseudomonas aeruginosa, Acinetobacter baumannii*, and *Stenotrophomonas maltophilia*. The expressions of metallo-β-lactamases such as inosine 5'-monophosphate (IMP), Verona integron-mediated metallo-β-lactamase (VIM), and New Delhi metallo**-**β-lactamase (NDM) are associated with a higher resistance rate of CFDC. Carbapenem-resistant phenotype has little effect on the resistance rate, although the acquisition of a particular carbapenemase may affect the susceptibility of the pathogens to CFDC. For potential resistance mechanism, mutations in β-lactamases and TonB-dependent receptors, which assist CFDC entering bacteria, would increase a minimum inhibitory concentration (MIC)90 value of CFDC against MDR pathogens. Since the development of CFDC, resistance during its utilization has been reported thus, prudent clinical applications are still necessary to preserve the activity of CFDC.

## Introduction

As an ongoing challenge to global health, the emergence of antibiotic-resistant infections results in substantial morbidity and mortality (1). Gram-negative bacteria (GNB) are increasingly associated with high rates of antimicrobial resistance, especially for the carbapenem-resistant *Enterobacteriaceae* (CRE), multidrug-resistant (MDR) *Acinetobacter baumannii* (*A. baumannii*), *Klebsiella pneumoniae* (*K. pneumoniae*), and *Pseudomonas aeruginosa* (*P. aeruginosa*) (2). Due to the current limited options of MDR pathogen-caused infections and the resistance for “cunning bacterias” to drugs, new therapeutic options are of particular concern and urgently necessary (3–5).

**Figure 1:**
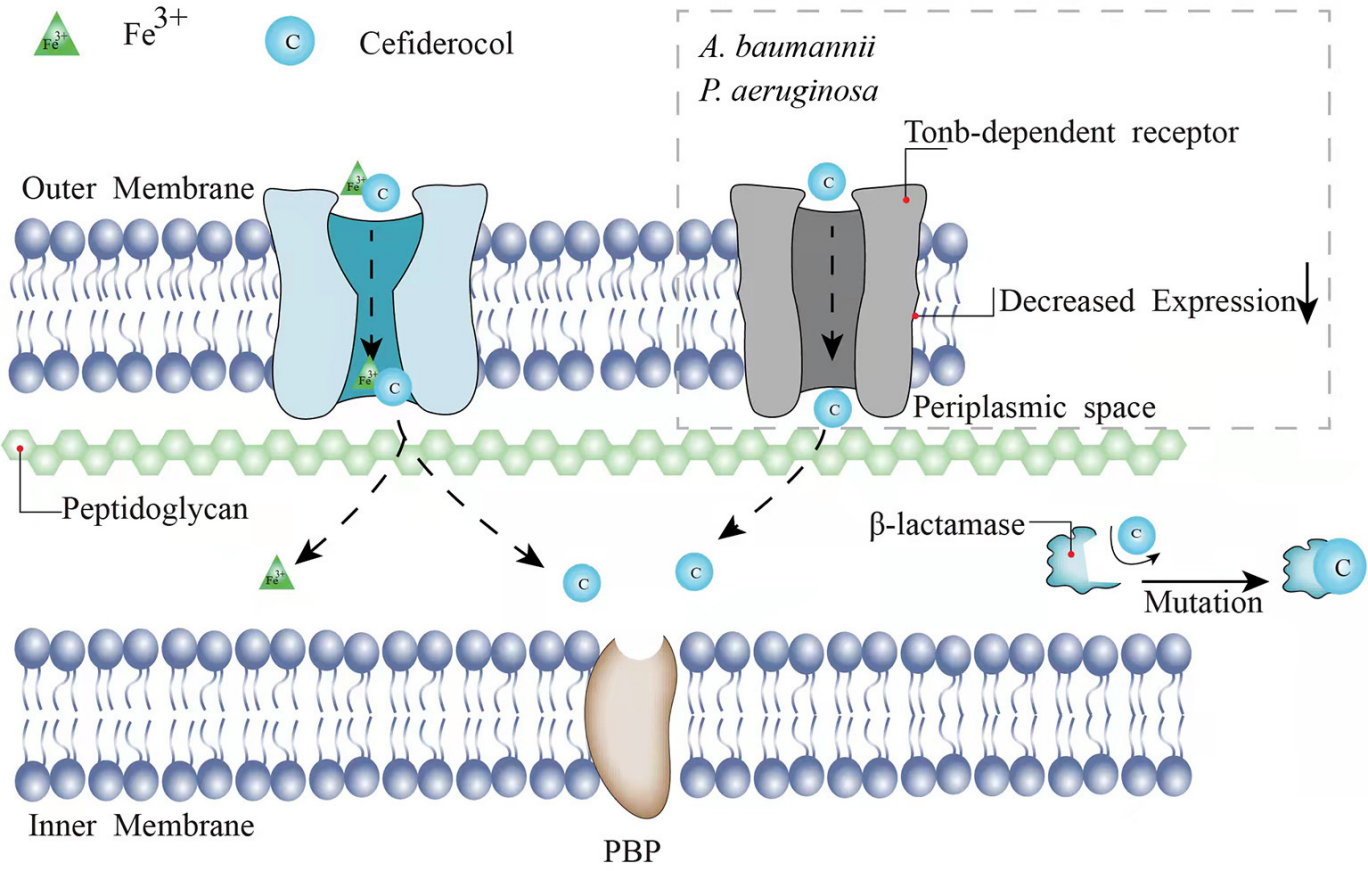
The mechanisms and underlying resistance mechanisms of CFDC.

As a novel injectable siderophore cephalosporin, cefiderocol (CFDC) has been approved by the United States Food and Drug Administration (FDA) for the treatment of complicated urinary tract infections (cUTIs) in 2019, hospital-acquired bacterial pneumonia (HABP), and ventilator-associated bacterial pneumonia (VABP) caused by GNB in 2020. In a study consisting of 377 patients with GNB-induced cUTI, 73% of 252 patients in the CFDC group was cured according to clinical response and microbiological response compared with the imipenem-cilastatin group (55% of 119 patients), indicating the good activity of CFDC (6). Same as other cephalosporins, the principal mechanism of CFDC is the inhibition of the cell wall by combining with penicillin-binding protein-3 (PBP-3), which the affinities [50% inhibitory concentrations (IC50s)] of cefiderocol for PBP-3 of *Escherichia coli* (*E. coli*) NIHJ JC-2, *K. pneumoniae* SR22291, *P. aeruginosa* ATCC 27853, and *A. baumannii* ATCC 17978 were 0.04 to 0.67 μg/ml (7). However, CFDC is more stable to β-lactamases because of its “Trojan horse” strategy (7). CFDC combines a cephalosporin core and a catechol-type siderophore, which are highly effective to acquire bacterial iron (Fe^3+^). Through binding to bacterial iron transporter outer membrane protein, CFDC can enter the bacterial periplasmic space to avoid the degradation of β-lactamase produced by the pathogen (7, 8) (Figure 1). Therefore, it shows activity against GNB pathogens including extended-spectrum β-lactamases (ESBL)-producing GNB, CRE, *P. aeruginosa, A. baumannii, K. pneumoniae, Klebsiella oxytoca*, and *Stenotrophomonas maltophilia* (*S. maltophilia*) (9–19). *Serratia marcescens, Citrobacter koseri, Burkholderia cepacia* (*B. cepacia*), and *Citrobacter freundii* (*C. freundii*) are also sensitive to CFDC with a minimum inhibitory concentration (MIC)90 value of under 1 mg/l (9, 11, 14–16, 19–21). The breakpoints of CFDC have been interpreted by different standards including the Clinical and Laboratory Standards Institute (CLSI), the FDA, and the European Committee on Antimicrobial Susceptibility Testing (EUCAST). The breakpoints of CLSI are commonly used and available for *Enterobacteriaceae, A. baumannii, P. aeruginosa*, and *S. maltophilia* (susceptible ≤ 4 mg/l, intermediate 8 mg/l, and resistant ≥16 mg/l). The breakpoints of the EUCAST for *Enterobacteriaceae* and *P. aeruginosa* are susceptible ≤ 2 mg/l and resistant >2 mg/l (22). The FDA breakpoints for *Enterobacteriaceae* have been changed from (susceptible ≤ 2 mg/l, intermediate 4 mg/l, and resistant ≥8 mg/l) in 2019 to (susceptible ≤ 4 mg/l, intermediate 8 mg/l, and resistant ≥16 mg/l) in 2020 and the standard for *A. baumannii* (susceptible ≤ 1 mg/l and resistant ≥4 mg/l) has been added. The breakpoints for *P. aeruginosa* remain as (susceptible ≤ 1 mg/l and resistant ≥4 mg/l). Broth microdilution and disk diffusion methods are both available for different standards.

Although it has not been long since CFDC appears in the market and its indications are limited, CFDC is highly anticipated and acts as a new option for the treatment of various MDR pathogens (23). Recently, a randomized controlled phase-3 trial study has reported that CFDC has similar clinical efficacy to the best available therapy in infections caused by carbapenem-resistant GNB (24). Another study has reported that CFDC is non-inferior to high-dose and extended-infusion meropenem in the treatment of MDR GNB infections (25).

In this study, we aim to review the *in-vitro* and *in-vivo* activity of CFDC to evaluate its global effectiveness so far (Supplementary Table 1) and to discuss the potential mechanism of CFDC resistance.

## Reports of *in-vitro* Resistance to CFDC

### Resistance Rate in *Enterobacterales*

Generally, CFDC has a high activity against *Enterobacterales* with most of the MIC90 values ≤ 8 mg/l (10, 11, 13–16, 18–21, 26, 27). Mariana et al. have reported a resistance rate of 5% for 335 *Enterobacterales* isolates according to the CLSI breakpoints, which are originated from the United States, Canada, and Singapore, with an MIC50 value ranging from 0.015 to >64 mg/l and an MIC90 value of 8 mg/l (21). However, the resistance rate shows obvious differences according to different phenotypes of β-lactamases. Based on the Ambler Classification system, β-lactamases are divided into four classes as follows: classes A, C, and D of serine β-lactamases and class B known as metallo-β-lactamases (MBLs). The β-lactamases that confer the reduction of drug sensitivity in *Enterobacterales* belong to the abovementioned types: two serine-β-lactamase including ESBL and *K. pneumoniae* carbapenemases (KPCs) and class B [MBL, especially New Delhi metallo-β-lactamases (NDM)]. Several reports have shown that the resistance rate of MBL-producing *Enterobacterales* is higher compared with non-MBL-producing pathogens (10, 28, 29). Two studies from Europe and the United Kingdom in 2020 have reported that the resistance rate of NDM-positive strains against CFDC is up to 59 and 48.6% (the EUCAST breakpoints), respectively, with an MIC90 value reaching 32 mg/l (10, 28). Two other studies have also reported the widest MIC90 range of CFDC against NDM-positive *Enterobacteriaceae* up to >64 mg/l (3) or 8 mg/l (30) compared with other β-lactamase phenotypes. Following NDM, another type of Verona integron-mediated MBL (VIM)-positive strain exhibits a high resistance of 19.1 and 21%, respectively (10, 28). The resistance rate of CFDC against class A β-lactamase-positive strains and carbapenemases, such as GES, IMI, SME, and TEM enzyme, is relatively lower compared with class B β-lactamase-positive strains (3, 10, 28). An MIC90 value of CFDC against ESBL + porin loss *Enterobacterales* ranges from 0.125 to 32 mg/l with a resistance rate of 38.5% (28). Studies from Europe have shown that the resistance rate of KPC-producing pathogens to CFDC is 8.9 and 16.4%, respectively, based on the EUCAST breakpoints (10, 28). Similarly, class D β-lactamase OXA-48-positive *Enterobacterales* show a resistance rate of 7.1 and 11.8%, respectively, with an MIC90 value reaching 8 mg/l (10, 28). However, another study from North America and Europe has shown that all the 75 KPC-positive and 32 OXA-48-positive strains are sensitive to CFDC (30). The class C β-lactamase AmpC-positive strains are susceptible to CFDC with the resistance rate of 0% (28). Meredith et al. have shown that the resistance rates of meropenem non-susceptible (MIC90 ≥2 mg/l) strains are all susceptible according to the CLSI breakpoints (19).

For *E. coli*, most of the reports have shown that an MIC90 value of CFDC is ≤ 4 mg/l ranging from 0.25 to 4 mg/l (9, 11, 13, 14, 16, 18–21, 29). Two studies have compared the MIC90 values between isolates from Europe and North America, showing that an MIC90 value of CFDC to the strains from Europe is twice higher compared with those from North America (16, 19), although all the stains are susceptible. Class B β-lactamase-positive *E. coli* has the highest resistance to CFDC (27, 29). Naoki et al. have reported that 26.3% of 19 isolates from NDM-1-producing *E. coli* are resistant to CFDC according to the CLSI breakpoints (29). The MIC90 values of CFDC against NDM (-1/4/5/6/7), VIM (-1/2/4/19), or inosine 5'-monophosphate (IMP) (-1/8) *E. coli* are significantly higher compared with other β-lactamase phenotypes isolates such as KPC (-2/3) or OXA-48 type (16 vs. 1 or 0.5 mg/l) (27).

For *K. pneumoniae*, an MIC90 value of CFDC is mostly ≤ 8 mg/L (9, 11, 13–16, 18, 21, 27, 29). In 2018, 689 carbapenem non-susceptible strains from North America and Europe are mostly susceptible, with an MIC90 value of 4 mg/l (18). However, C Paul et al. have reported that the resistance rate of 15 carbapenem-resistant *K. pneumoniae* is up to 20% based on the CLSI breakpoints, with an MIC90 value of 32 mg/l (20). Kenneth et al. have also reported the widest MIC90 range >64 mg/l and a resistance rate of 4.3% of CFDC against carbapenem-resistant *K. pneumoniae* according to the CLSI breakpoints (9). Different phenotypes also affect the sensitivity of *K. pneumoniae*. An MIC90 (4 mg/l) value of CFDC against MBL-positive strains is twice or four times higher than that of KPC- or OXA-48-positive strains (27). For class A β-lactamases, the resistance rate of CFDC against 23 isolates from ESBL-producing *K. pneumoniae* is 2.7%, with an MIC90 value ranging from 0.125 to >64 mg/l (9). However, the other two studies both found that ESBL-positive isolates were sensitive to CFDC, with the highest MIC90 value of 4 mg/l (14, 29). The KPC (−2/3/11) and OXA (-48/162/163/181/204/232) isolates are also susceptible to CFDC with an MIC90 value <4 mg/l (15, 27, 29). Besides, the same genus of bacteria may have different MICs when large samples of detection conducted by different regions. James et al. have reported that an MIC90 value of CFDC to the strains from Europe (2 mg/l) is four times higher compared with those from North America (0.5 mg/l) (16).

For other *Enterobacterales*, the decreased sensitivity is mainly attributed to the production of OXA or MBLs (NDM, VIM, or IMP), with an MIC90 value (4 mg/l) four times higher compared with KPC-positive strains (27). An MIC90 value of CFDC against carbapenem-resistant pathogens is also increased up to 8 mg/l, ranging from 0.06 to 32 mg/l (18). *Enterobacter cloacae* (*E. cloacae*) is susceptible to CFDC with an MIC90 value ≤ 1 mg/l (11, 14, 16, 29). According to the CLSI breakpoints, C Paul et al. have reported that the resistance rate of carbapenem-resistant *E. cloacae* complex is 13%, with an MIC90 value of 16 mg/l (20). The resistance rate of CFDC against 38 ESBL-positive strains is 5.3% using the CLSI breakpoints, with an MIC90 value ranging from < 0.03 to >64 mg/l (9). CFDC shows excellent activity against nonenzyme-producing *Klebsiella spp., Serratia spp., Citrobacter spp*., and *Proteus mirabilis* with a resistance rate of 0% (9, 11, 14, 16, 18–20, 29). Carbapenem non-susceptibility is the main factor for the decreased MIC90 value of *Klebsiella spp., Serratia spp*., and *Citrobacter spp*. and will result in a two- or four-time increase of CFDCs MIC90 (18, 20). Although an MIC90 value of *C. freundii* ranges from ≤ 0.063 to >64 mg/l and the resistance rate is not provided by Naoki et al., an MIC90 value is as low as 0.125 mg/l (29).

### Pseudomonas aeruginosa

Generally, CFDC shows an excellent bactericidal effect against *P. aeruginosa* with an MIC90 value of ≤ 2 mg/l (3, 13–19, 27, 30). MBLs are still correlated to their CFDC resistance. A study from the United Kingdom has reported that the resistance rate of isolates from 11 NDM-positive and 30 IMP-positive *P. aeruginosa* is 54.5 and 20%, respectively, according to the EUCAST breakpoints, with the upper range of an MIC90 value of ≥ 128 mg/l (28). The resistance rate of the class A β-lactamase (GES, PER, and VEB)-producing isolates is relatively high at 10–33.3% (28). However, two European studies have reported that the resistance rates of VIM-, IMP-, NDM-, and GES-positive *P. aeruginosa* are all 0% (10, 30). Although Dobias et al. did not provide the resistance rate against CFDC, an MIC90 value of 2 mg/l reflects the high activity of CFDC against VIM-, IMP-, KPC-, SPM-, or GIM-producing *P. aeruginosa* (27). An MIC90 value of CFDC against carbapenemase-producing *P. aeruginosa* is a little bit higher compared with non-carbapenemase ones (2 vs. 0.5 mg/l) in a German study, with a resistance rate of 9.1 and 0%, respectively (11). A study from the USA has also reported a high MIC90 value of 8 mg/l in carbapenem-resistant strains (20). The activity of CFDC against carbapenem non-susceptible or MDR *P. aeruginosa* remains well, with an MIC90 value of ≤ 2 mg/l (14, 17, 19, 30).

### *Acinetobacter* spp.

Most studies have demonstrated that an MIC90 value of CFDC against non-enzymes-producing *A. baumannii* is <4 mg/l (3, 10, 13, 15, 16, 19, 27, 30, 31). Ceftazidime resistant had little effect on an MIC90 value of CFDC to *A. baumannii* (13). However, one study has reported that an MIC90 value of CFDC to 97 *A. baumannii* isolates is 32 mg/l according to the CLSI breakpoints, with a resistance rate of 33%. The pathogens collected from the United States, Canada, and Singapore from 1996 to 2015 possess one or multiple types of gene expression including *bla*_CMY_, *bla*_CTX−M_, *bla*_FOX_, *bla*_IMI_, *bla*_IMP_, *bla*_KPC_, *bla*_NDM_, *bla*_OXA−48−like_, *bla*_SHV_, *bla*_SME_, and *bla*_TEM_ (21). *A. baumannii* resistance is attributed to the production of OXA- or NDM-type enzymes. Moreover, the resistance rate varies according to different phenotypes of OXA enzymes. The resistance rate of CFDC against OXA-23-positive *A. baumannii* is 14.6%, while it is 11.1, 10, and 5.3% for OXA-24/40-positive strains, OXA-58-positive strains, and OXA-51-positive strains, respectively, using non-species special pharmacokinetic-pharmacodynamic (PK-PD) breakpoints (22, 28). Akinobu et al. have demonstrated that the resistance rate of CFDC against OXA-23-positive strains (16.7%) is relatively higher compared with other phenotypes of OXA-positive *A. baumannii* (0%), with the maximum range of an MIC90 value of >32 mg/l (31). Iregui et al. have also reported that the resistance rate of *bla*_OXA−23_ *A. baumannii* is 8.8% according to the CLSI breakpoints, with an MIC90 value of 8 mg/l (13). However, Delgado et al. from Spain and Christopher et al. from Europe have reported that the resistance rate of OXA-24/40-positive strains is 12 and 6.8%, respectively, which is higher compared with other phenotypes (10, 15). A study from the United Kingdom has demonstrated that 20 NDM-producing pathogens show the highest resistance of 50% based on non-species special PK-PD breakpoints, with an MIC90 value ranging from 1 to ≥128 mg/l (22, 28) (since no MIC90 criteria have been provided for CFDC to *A. baumannii*). A Chinese study has reported that the resistance rate of imipenem-resistant pathogens is 7% in 2020 using the CLSI breakpoints, with an MIC90 value ranging from 0.06 to >64 mg/l and an MIC90 value of 8 mg/l (17). Other studies have also shown that carbapenem-resistant strains are more resistant to CFDC compared with the susceptible strains, with a slightly higher MIC90 value or MIC90 range (3, 19, 31). MDR *A. baumannii* exhibits the highest resistance with an MIC90 value of 8 mg/l and an MIC90 range reaching >256 mg/l (18). Two studies have compared an MIC90 value between the isolates from Europe and North America and no significant difference has been found (16, 19).

According to the CLSI breakpoints, Kenneth et al. have shown that the resistance rate of *Acinetobacter spp*. is 10%, with an MIC90 value of 4 mg/l (9). However, CFDC has high activity against *Acinetobacter pittii* from North America and Europe, reported by James et al., with the resistance rate of 0% and an MIC90 value of 0.5 mg/l (16).

### Other Strains

For *S. maltophilia, B. cepacia complex, Morganellaceae, Achromobacter xylosoxidans*, and *Proteus mirabilis*, they show excellent susceptibility to CFDC, with a resistance rate of 0% and an MIC90 value of <1 mg/l, as reported by worldwide studies (11, 14–21, 26).

## Reports of *in-vivo* Resistance to CFDC

Several animal studies demonstrated that strains carring KPC or NDM may reduce the suscepitbility to CFDC. An *in-vivo* study using neutropenic murine thigh and lung infection models has shown that an MIC90 of NDM-producing GNB including *E. coli, K. pneumoniae*, and *P. aeruginosa* was 8- to 64-fold higher than non-producing strains (32). The MIC90 of NDM-1-producing *K. pneumoniae* sequence type 14 (ST14) reach to 16 mg/l, which is resistant to CFDC (32). The MIC90 of KPC-producing pathogens is 16 times higher than non-producing *K. pneumoniae* (4 vs. 0.25 mg/l) (32). In a immunocompetent rat respiratory tract infection model, an MIC90 value at 8 mg/l of NDM-1-positive *K. pneumoniae* is two times higher than KPC-2-positive *K. pneumoniae* (33). In *in-vivo* models, the amount of inoculation will also affect the MIC90 of CFDC. An MIC90 value at 16–64 mg/l was observed in a *K. pneumoniae* infected neutropenic murine thigh model, which was infected with 10^7^ CFU/ml bacterial suspension (34). According to the EUCAST breakpoints, Hobson et al. have also reported that high inocula (10^7^ CFU/ml) with KPC-producing *Enterobacteriaceae* will lead the resistance to CFDC compared to usual inocula (10^5^ CFU/ml) in 2021 (35).

Grande et al. have reported that a 63-year-old male patient with septic shock is presented at the intensive care unit (ICU) due to the initial infection of ESBL *K. pneumoniae*, oxacillin-sensitive *Staphylococcus aureus*, and multi-sensitive *P. aeruginosa*. Then, a VIM-producing XDR *P. aeruginosa* grows from his sputum on day 26 (36). On day 54, the regimen of colistin and meropenem is switched to CFDC 2 g q8h infused over 3 h plus metronidazole 500 mg TID as *P. aeruginosa* is susceptible to CFDC. The treatment regimen is discontinued after 6 weeks (36). On day 128, GES- and VIM-producing XDR *P. aeruginosa* are isolated from ischial eschar with an MIC90 value of CFDC increased to 8 mg/l (36). It indicates that *P. aeruginosa* develops resistance during CFDC treatment.

## Resistance Mechanisms

Previous studies have shown that the presence of single-type MBLs may increase an MIC90 value of CFDC against part of the isolates. However, one study has demonstrated that the co-expression of MBLs and serine-type β-lactamases is related to the non-susceptibility of CFDC (37). An MIC90 value to CFDC presents an 8- to 64-fold and 8-fold reduction against CFDC-resistant *Enterobacterales* (including *E. coli* and *K. pneumoniae*) and *A. baumannii*, respectively, when both the dipicolinic acid (an MBL inhibitor) and avibactam (a serine-β-lactamase inhibitor) are added to the susceptible level (≤0.5 μg/ml) (37). However, an MIC90 value of ≤ 2-fold for CFDC has not been observed by the addition of dipicolinic acid or avibactam alone (37). Mutations in β-lactamases may also lead to CFDC resistance (Figure 1). A 4- to 32-fold increase of an MIC90 was observed in D179Y-H274Y mutations of KPC-31 compared to the wild-type alleles reported by Hobson et al. in 2021 (35). Shields et al. have reported that the deletion of positions 292 and 293, which are located in the R2 loop of AmpC, causes the decreased susceptibility of *Enterobacterales* (38). The mutations lead to the disappearance of the H10 helix in the R2 loop and the expansion of the substrate-binding site, resulting in a more stable binding to the bulkier side chain possessed by CFDC (38). Akito et al. have demonstrated the alanine-proline deletion at positions 294 and 295 located in the R2 loop, which is also associated with the reduced susceptibility to CFDC in *E. coli and E. cloacae* (39). Especially for *E. cloacae*, the depletion of A294_L295 results in an increase of >32-fold in an MIC90 value of CFDC (39).

TonB-dependent receptors commonly exist in GNB outer membrane and assist CFDC to enter the bacterial periplasmic space *via* cooperation with the TonB-ExbB-ExbD complex located in the cytoplasmic membrane (40). The energy required for the transport of CFDC is provided by TonB-ExbB-ExbD complex (40). The main TonB-dependent receptors of *A. baumannii* are termed as PiuA and PirA (41). Malik et al. have reported that the change from a hydrophobic amino acid to an aromatic amino acid at location 275 of PirA and the downregulation of *pirA*, possibly in combination with loss of *piuA*, cause the decreased expressions of TonB-dependent receptors, which interpret the increased resistance to CFDC in *A. baumannii* (42) (Figure 1). Decreased sensitivity to CFDC has also been observed in *P. aeruginosa* when the loss of *piuA* and downregulation of TonB-dependent receptors occur (41) (Figure 1). Alexandre et al. have shown that the decreased expression of PiuA ortholog, termed as PiuD, which is encoded by *piuD*, is more important than the loss of *piuA* (43). They had tested an MIC90 value of *P. aeruginosa* when *piuA* or *piuD* is depleted and found that the deletion of *piuA* increases the CFDC MIC90 value by 2-fold, while such elevation for the deletion of *piuD* is 32-fold (43). Moynié et al. have considered that TonB3-ExbB3-ExbD3 complex not only provides energy for the siderophore transport, but also is associated with siderophore acquiring Fe^3+42^. Mutation of TonB3-ExbB3-ExbD3 by insertion of A at position 9 in the *exbD3* gene, deletion of A at position 319, and insertion of A at position 243 in the *tonB3* gene would impede energy acquisition for transport and iron availability. Therefore, the transmission of CFDC to bacteria would be inhibited (41).

## Conclusion

In conclusion, CFDC has demonstrated excellent activity against GNB including ESBL *Enterobacterales*, CRE, MDR *A. baumannii*, and carbapenem-resistant *P. aeruginosa*. The expressions of MBLs are associated with the decreased sensitivity of pathogens to CFDC. However, the acquisition of a particular β-lactamase does not ensure resistance and additional mechanisms such as mutations in β-lactamases are necessary for overt resistance to develop. Moreover, since the CFDC resistance has already been reported during its anti-infective therapy, the clinical application needs to be cautious to preserve the activity of CFDC.

## Author Contributions

JY wrote the first draft of the manuscript. JW, MC, and YC contributed to manuscript revision. All authors contributed to the article and approved the submitted version.

## Funding

This study was supported by the National Natural Science Foundation of China (81770004 and 82073894) and the Cultivation Project of PLA General Hospital for Distinguished Young Scientists (2020-JQPY-004).

## Conflict of Interest

The authors declare that the research was conducted in the absence of any commercial or financial relationships that could be construed as a potential conflict of interest.

## Publisher's Note

All claims expressed in this article are solely those of the authors and do not necessarily represent those of their affiliated organizations, or those of the publisher, the editors and the reviewers. Any product that may be evaluated in this article, or claim that may be made by its manufacturer, is not guaranteed or endorsed by the publisher.

## References

[1] Bart S Rubin D Kim P Farley J Nambiar S. Trends in hospital-acquired and ventilator-associated bacterial pneumonia trials. Clin Infect Dis. (2020) 2020:ciaa1712. 10.1093/cid/ciaa1712 33173946

[2] Karakonstantis S Kritsotakis EI Gikas A. Treatment options for pneumoniae K, *P. aeruginosa* and *A. baumannii* co-resistant to carbapenems, aminoglycosides, polymyxins and tigecycline: an approach based on the mechanisms of resistance to carbapenems. Infection. (2020) 48:835–51. 10.1007/s15010-020-01520-6 32875545 PMC7461763

[3] Jacobs MR Abdelhamed AM Good CE Rhoads DD Hujer KM Hujer AM. ARGONAUT-I: activity of cefiderocol (S-649266), a siderophore cephalosporin, against gram-negative bacteria, including carbapenem-resistant nonfermenters and enterobacteriaceae with defined extended-spectrum β-lactamases and carbapenemases. Antimicrob Agents Chemother. (2019) 63:18. 10.1128/AAC.01801-18 30323050 PMC6325197

[4] Wang Y Wang J Wang R Cai Y. Resistance to ceftazidime-avibactam and underlying mechanisms. J Glob Antimicrob Resist. (2020) 22:18–27. 10.1016/j.jgar.2019.12.009 31863899

[5] Chen J Zeng Y Zhang R Cai J. *In vivo* emergence of colistin and tigecycline resistance in carbapenem-resistant hypervirulent *Klebsiella pneumoniae* during antibiotics treatment. Front Microbiol. (2021) 12:702956. 10.3389/fmicb.2021.702956 34603229 PMC8482011

[6] Portsmouth S van Veenhuyzen D Echols R Machida M Ferreira JCA Ariyasu M. Cefiderocol vs. imipenem-cilastatin for the treatment of complicated urinary tract infections caused by Gram-negative uropathogens: a phase 2, randomised, double-blind, non-inferiority trial. Lancet Infect Dis. (2018) 18:1319–28. 10.1016/S1473-3099(18)30554-1 30509675

[7] Ito A Sato aT Ota aM Takemura aM Nishikawa aT. *In vitro* antibacterial properties of cefiderocol, a novel siderophore cephalosporin, against Gram-negative bacteria. Antimicrob Agents Chemother. (2018) 62:e01454–17. 10.1128/AAC.01454-17 29061741 PMC5740388

[8] Aoki T Yoshizawa H Yamawaki K Yokoo K Sato J Hisakawa S. Cefiderocol (S-649266), a new siderophore cephalosporin exhibiting potent activities against *Pseudomonas aeruginosa* and other gram-negative pathogens including multi-drug resistant bacteria: structure activity relationship. Eur J Med Chem. (2018) 155:847–68. 10.1016/j.ejmech.2018.06.014 29960205

[9] Rolston KVI Gerges B Shelburne S Aitken SL Raad I Prince RA. Activity of cefiderocol and comparators against isolates from cancer patients. Antimicrob Agents Chemother. (2020) 64:19. 10.1128/AAC.01955-19 32071053 PMC7179642

[10] Longshaw C Manissero D Tsuji M Echols R Yamano Y. *In vitro* activity of the siderophore cephalosporin, cefiderocol, against molecularly characterized, carbapenem-non-susceptible Gram-negative bacteria from Europe. JAC-Antimicrobial Resistance. (2020) 2:dlaa060. 10.1093/jacamr/dlaa060 34223017 PMC8210120

[11] Kresken M Korte-Berwanger M Gatermann SG Pfeifer Y Pfennigwerth N Seifert H. *In vitro* activity of cefiderocol against aerobic Gram-negative bacterial pathogens from Germany. Int J Antimicrob Agents. (2020) 56:106128. 10.1016/j.ijantimicag.2020.106128 32758648

[12] Johnston BD Thuras P Porter SB Anacker M VonBank B Snippes Vagnone P. Activity of cefiderocol, ceftazidime-avibactam, and eravacycline against carbapenem-resistant *Escherichia coli* isolates from the United States and International Sites in Relation to Clonal Background, Resistance Genes, Coresistance, and Region. Antimicrob Agents Chemother. (2020) 64:20. 10.1128/AAC.00797-20 32718965 PMC7508590

[13] Iregui A Khan Z Landman D Quale J. Activity of cefiderocol against enterobacterales, *Pseudomonas aeruginosa*, and *Acinetobacter baumannii* endemic to medical centers in New York City. Microbial Drug Resistance. (2020) 26:722–6. 10.1089/mdr.2019.0298 32031915 PMC7368386

[14] Golden AR Adam HJ Baxter M Walkty A Lagacé-Wiens P Karlowsky JA Zhanel GG. *In vitro* activity of cefiderocol, a novel siderophore cephalosporin, against gram-negative bacilli isolated from patients in Canadian Intensive Care Units. Diagn Microbiol Infect Dis. (2020) 97:115012. 10.1016/j.diagmicrobio.2020.115012 32081522

[15] Delgado-Valverde M Conejo MDC Serrano L Fernández-Cuenca F Pascual Á. Activity of cefiderocol against high-risk clones of multidrug-resistant *Enterobacterales, Acinetobacter baumannii, Pseudomonas aeruginosa* and *Stenotrophomonas maltophilia*. J Antimicrob Chemother. (2020) 75:1840–9. 10.1093/jac/dkaa117 32277821 PMC7303814

[16] Karlowsky JA Hackel MA Tsuji M Yamano Y Echols R Sahm DF. *In vitro* activity of cefiderocol, a siderophore cephalosporin, against gram-negative bacilli isolated by clinical laboratories in North America and Europe in 2015-2016: SIDERO-WT-2015. Int J Antimicrob Agents. (2019) 53:456–66. 10.1016/j.ijantimicag.2018.11.007 30471402

[17] Hsueh SC Lee YJ Huang YT Liao CH Tsuji M Hsueh PR. *In vitro* activities of cefiderocol, ceftolozane/tazobactam, ceftazidime/avibactam and other comparative drugs against imipenem-resistant *Pseudomonas aeruginosa* and *Acinetobacter baumannii*, and *Stenotrophomonas maltophilia*, all associated with bloodstream infections in Taiwan. J Antimicrob Chemother. (2019) 74:380–6. 10.1093/jac/dky425 30357343

[18] Hackel MA Tsuji M Yamano Y Echols R Karlowsky JA Sahm DF. *In vitro* activity of the siderophore cephalosporin, cefiderocol, against carbapenem-nonsusceptible and multidrug-resistant isolates of gram-negative bacilli collected worldwide in 2014 to 2016. Antimicrob Agents Chemother. (2018) 62:17. 10.1128/AAC.01968-17 29158270 PMC5786755

[19] Hackel MA Tsuji M Yamano Y Echols R Karlowsky JA Sahm DF. *In vitro* activity of the siderophore cephalosporin, cefiderocol, against a recent collection of clinically relevant gram-negative bacilli from North America and Europe, including carbapenem-nonsusceptible isolates (SIDERO-WT-2014 study). Antimicrob Agents Chemother. (2017) 61:17. 10.1128/AAC.00093-17 28630181 PMC5571285

[20] Morris CP Bergman Y Tekle T Fissel JA Tamma PD Simner PJ. Cefiderocol antimicrobial susceptibility testing against multidrug-resistant gram-negative bacilli: a comparison of disk diffusion to broth microdilution. J Clin Microbiol. (2020) 59:20. 10.1128/JCM.01649-20 32938734 PMC7771458

[21] Albano M Karau MJ Schuetz AN Patel R. Comparison of agar dilution to broth microdilution for testing *in vitro* activity of cefiderocol against gram-negative bacilli. J Clin Microbiol. (2020) 2020:20. 10.1128/JCM.00966-20 32967901 PMC7771473

[22] EUCAST. Breakpoints for Cefiderocol From EUCAST. Addendum (May 20202) to EUCAST Breakpoint Tables V.10.0. Breakpoints to be Included in EUCAST Breakpoint Tables v 11.0, January 2021. (2020). Available online at: https://yc.mlpla.mil.cn/s/org/eucast/www/G.https/fileadmin/src/media/PDFs/EUCAST_files/Breakpoint_tables/Addenda/Cefiderocol_addendum_20200501.pdf

[23] Wu JY Srinivas P Pogue JM. Cefiderocol: a novel agent for the management of multidrug-resistant gram-negative organisms. Infect Dis Ther. (2020) 9:17–40. 10.1007/s40121-020-00286-6 32072491 PMC7054475

[24] Bassetti M Echols R Matsunaga Y Ariyasu M Doi Y Ferrer R. Efficacy and safety of cefiderocol or best available therapy for the treatment of serious infections caused by carbapenem-resistant Gram-negative bacteria (CREDIBLE-CR): a randomised, open-label, multicentre, pathogen-focused, descriptive, phase 3 trial. Lancet Infect Dis. (2020) 2020:9. 10.1016/S1473-3099(20)30796-9 33058795

[25] Wunderink RG Matsunaga Y Ariyasu M Clevenbergh P Echols R Kaye KS. Cefiderocol vs. high-dose, extended-infusion meropenem for the treatment of Gram-negative nosocomial pneumonia (APEKS-NP): a randomised, double-blind, phase 3, non-inferiority trial. Lancet Infect Dis. (2020) 2020:3. 10.1016/S1473-3099(20)30731-3 33058798

[26] Jacobs MR Abdelhamed AM Good CE Rhoads DD Hujer KM Hujer AM. *In vitro* activity of cefiderocol (s-649266), a siderophore cephalosporin, against enterobacteriaceae with defined extended-spectrum b-lactamases and carbapenemases. Open ForInfect Dis. (2018) 5:S413–4. 10.1093/ofid/ofy210.1182 PMC6325197 30323050

[27] Dobias J Dénervaud-Tendon V Poirel L Nordmann Nordmann P Activity of the novel siderophore cephalosporin cefiderocol against multidrug-resistant Gram-negative pathogens. Eur J Clin Microbiol Infect Dis. (2017) 36:2319–27. 10.1007/s10096-017-3063-z 28748397

[28] Mushtaq S Sadouki Z Vickers A Livermore DM Woodford N. *In vitro* activity of cefiderocol, a siderophore cephalosporin, against multidrug-resistant gram-negative bacteria. Antimicrobial Agents Chemotherapy. (2020) 64:20. 10.1128/AAC.01582-20 32958717 PMC7674041

[29] Kohira N West J Ito A Ito-Horiyama T Nakamura R Sato T. *In vitro* antimicrobial activity of a siderophore cephalosporin, s-649266, against enterobacteriaceae clinical isolates, including carbapenem-resistant strains. Antimicrob Agents Chemother. (2016) 60:729–34. 10.1128/AAC.01695-15 26574013 PMC4750680

[30] Kazmierczak KM Tsuji M Wise MG Hackel M Yamano Y Echols R. *In vitro* activity of cefiderocol, a siderophore cephalosporin, against a recent collection of clinically relevant carbapenem-non-susceptible Gram-negative bacilli, including serine carbapenemase- and metallo-β-lactamase-producing isolates (SIDERO-WT-2014 Study). Int J Antimicrob Agents. (2019) 53:177–84. 10.1016/j.ijantimicag.2018.10.007 30395986

[31] Ito A Kohira N Bouchillon SK West J Rittenhouse S Sader HS. *In vitro* antimicrobial activity of S-649266, a catechol-substituted siderophore cephalosporin, when tested against non-fermenting Gram-negative bacteria. J Antimicrob Chemother. (2016) 71:670–7. 10.1093/jac/dkv402 26645269

[32] Nakamura R Ito-Horiyama T Takemura M Toba S Matsumoto S Ikehara T. *In vitro* pharmacodynamic study of cefiderocol, a novel parenteral siderophore cephalosporin, in murine thigh and lung infection models. Antimicrobial Agents Chemother. (2019) 63:e02031–18. 10.1128/AAC.02031-18 31262762 PMC6709502

[33] Matsumoto S Singley CM Hoover J Nakamura R Echols R Rittenhouse S. Efficacy of cefiderocol against carbapenem-resistant gram-negative bacilli in immunocompetent-rat respiratory tract infection models recreating human plasma pharmacokinetics. Antimicrobial Agents Chemother. (2017) 61:e00700–17. 10.1128/AAC.00700-17 28630178 PMC5571323

[34] Monogue ML Tsuji M Yamano Y Echols R Nicolau DP. Efficacy of humanized exposures of cefiderocol (S-649266) against a diverse population of gram-negative bacteria in a murine thigh infection model. Antimicrobial Agents Chemother. (2017) 61:e01022–17. 10.1128/AAC.01022-17 28848004 PMC5655050

[35] Hobson CA Cointe A Jacquier H Choudhury A Magnan M Courroux C. Cross resistance to cefiderocol and ceftazidime-avibactam in KPC beta-lactamase mutants and inoculum effect. Clin Microbiol Infect. (2021) 2021:16. 10.1016/j.cmi.2021.04.016 33915286

[36] Grande Perez C Maillart E Miendje Deyi VY Huang TD Kamgang P Dernier Y. Compassionate use of cefiderocol in a pancreatic abscess and emergence of resistance. Médecine et Maladies Infectieuses. (2020) 2020:22. 10.1016/j.medmal.2020.10.022 33164837

[37] Kohira N Hackel MA Ishioka Y Kuroiwa M Sahm DF Sato T. Reduced susceptibility mechanism to cefiderocol, a siderophore cephalosporin, among clinical isolates from a global surveillance programme (SIDERO-WT-2014). J Glob Antimicrob Resist. (2020) 22:738–41. 10.1016/j.jgar.2020.07.009 32702396

[38] Shields RK Iovleva A Kline EG Kawai A McElheny CL Doi Y. Clinical evolution of AmpC-mediated ceftazidime-avibactam and cefiderocol resistance in *Enterobacter cloacae* complex following exposure to cefepime. Clin Infect Dis. (2020) 71:2713–6. 10.1093/cid/ciaa355 32236408 PMC7744991

[39] Kawai A McElheny CL Iovleva A Kline EG Sluis-Cremer N Shields RK. Structural basis of reduced susceptibility to ceftazidime-avibactam and cefiderocol in *Enterobacter cloacae* due to AmpC R2 loop deletion. Antimicrob Agents Chemother. (2020) 64:20. 10.1128/AAC.00198-20 32284381 PMC7318025

[40] Hartney SL Mazurier S Girard MK Mehnaz S Davis EW 2nd Gross H. Ferric-pyoverdine recognition by Fpv outer membrane proteins of Pseudomonas protegens Pf-5. J Bacteriol. (2013) 195:765–76. 10.1128/JB.01639-12 23222724 PMC3562110

[41] Moynié L Luscher A Rolo D Pletzer D Tortajada A Weingart H. Structure and function of the PiuA and PirA siderophore-drug receptors from *Pseudomonas aeruginosa* and *Acinetobacter baumannii*. Antimicrob Agents Chemother. (2017) 61:16. 10.1128/AAC.02531-16 28137795 PMC5365723

[42] Malik S Kaminski M Landman D Quale J. Cefiderocol resistance in *Acinetobacter baumannii*: roles of β-lactamases, siderophore receptors, and penicillin binding protein 3. Antimicrob Agents Chemother. (2020) 64:20. 10.1128/AAC.01221-20 32868330 PMC7577126

[43] Luscher A Moynié L Auguste PS Bumann D Mazza L Pletzer D. TonB-dependent receptor repertoire of *Pseudomonas aeruginosa* for uptake of siderophore-drug conjugates. Antimicrob Agents Chemother. (2018) 62:18. 10.1128/AAC.00097-18 29555629 PMC5971595

