# Supplementary material for: Cefiderocol: An Overview of Its in-vitro and in-vivo Activity and Underlying Resistant Mechanisms
Source: Front Med (Lausanne). 2021 Dec 7;8:741940. doi: 10.3389/fmed.2021.741940 (PMC8688709; doi:10.3389/fmed.2021.741940)
Supplement: Supplementary file 1 [file Table_1.pdf]

Table 1. Resistance rate of pathogens against CFDC

Year / Author	Region/ country and time of strain collection. Criteria for resistance determination	Pathogen	Characteristics of pathogen	R%* (No. of strains)[a]	MIC_{50} (mg/L)	MIC_{90} (mg/L)	MIC range (mg/L)
Kenneth[9] [2020]	Worldwide 2014-2017 CLSI 2019	*E. coli*	ESBL<br>CRE	0(52)<br>0(10)	-	2<br>4	0.3 to 4<br>0.6 to 8
		K. pneumoniae	ESBL<br>CRE	2.7(37)<br>4.3(23)	-	2	0.125 to >64<br>0.6 to >64
		E. cloacae	ESBL<br>CRE	5.3(38)<br>0(7)	-	4	<0.03 to >64<br>0.12 to 8
		Citrobacter spp.	-	0(20)	-	1	<0.03 to 8
		Serratia spp.	-	0(20)	-	0.5	<0.03 to 0.5
		P. aeruginosa	MDR	3.1(32)	-	1	<0.03 to >64
		Acinetobacter spp.	-	10(20)	-	4	<0.03 to >64
Brian[12] [2020]	Worldwide 2002-2017 FDA 2020	*E. coli*	CRE: 43%CTX-M, 28%CMY-2, 16% KPC, 13% OXA-48, 16%NDM, 2%IMP, 1%VIM	-(343)	0.5	2	≤0.004 to >64
Iregui[13] [2020]	New York 2013-2014,2017 CLSI 2020	*E. coli*	2017:141 ceftazidime-resistant	0(1869)	0.12	0.5	≤0.03 to 2
		Enterobacter spp.	2017: 58 *K. aerogenes*<br>104 *E. cloacae*(38 ceftazidime-resistant, 18 piperacillin/tazobactam-nonsusceptible)	0(172)	0.12	0.5	≤0.03 to 1

		K. pneumoniae	2013-2014: KPC<br>2017:19 *bla*$_{KPC}$	0(111)<br>0(517)	0.25<br>0.12	0.5	≤0.03 to 4<br>≤0.03 to 2
		A. baumannii	2013-2014: 47 *bla*$_{OXA-23}$<br>2017: 18 ceftazidime-resistant	8.8(78)<br>0(46)	0.5<br>0.25	8<br>1	0.12 to >32<br>0.06 to 4
		P. aeruginosa	2013-2014: CRE<br>2017: 33 *ampC*	0(130)<br>0(269)	0.5<br>0.25	1<br>0.5	≤0.03 to 4<br>≤0.03 to 8
Delgado[15]<br>[2020]	Spain<br>2014-2018<br>CLSI 2019	*E. cloacae*	2 CTX-M-15<br>1VIM-1; 1 OXA-48 +VIM-1	0(4)	-	-	0.5 to 8
		K. pneumoniae	25 ST11/OXA-48 + CTX-M-15<br>25 ST15/OXA-48 + CTX-M-15<br>25 ST512/KPC-3<br>25 ST258/KPC-3<br>3 ST147/OXA-48<br>4 ST392/OXA-48 + CTX-M-15	0(107)	0.25<br>0.25<br>2<br>2<br>0.25<br>0.25	2<br>4<br>4<br>2<br>0.5<br>1	≤0.03 to 4<br>≤0.03 to 4<br>0.25 to 4<br>0.06 to 4<br>0.06 to 0.5<br>0.06 to 1
		A. baumannii	25 ST2 / OXA-23<br>25 ST2 / OXA-24 / 40<br>25 ST2/OXA-58<br>5 ST745/OXA-58	0(25)<br>12(25)<br>0(25)<br>0(5)	0.25<br>2<br>0.125<br>-	0.5<br>16<br>0.5<br>-	0.06 to 1<br>0.5 to 16<br>0.06 to 0.5<br>0.06 to 0.25
		P. aeruginosa	5 non-carbapenemase, 1 IMP-16, CTX-M-15	0(6)	-	-	0.125 to 0.5
		S. maltophilia	-	0(20)	0.25	0.5	≤0.03 to 2
C. Paul[31]<br>[2020]	USA<br>2017<br>CLSI 2020<br>FDA 2019<br>EUCAST 2020	*E. coli*	Carbapenem-resistant	CLSI 0(15)<br>FDA 0(15)<br>EUCAST 0(15)	0.25	1	0.06 to 2
		K. pneumoniae	Carbapenem-resistant	CLSI 20(15)<br>FDA 20(15)<br>EUCAST 47(15)	2	32	0.06 to 32

		E. cloacae complex	Carbapenem-resistant	CLSI 13(15)<br>FDA 20(15)<br>EUCAST 40(15)	0.5	16	0.06 to 64
		K. aerogenes	Carbapenem-resistant	CLSI 0(2)<br>FDA 0(2)<br>EUCAST 0(2)	0.5	1	0.5 to 1
		K. oxytoca	Carbapenem-resistant	CLSI 0(6)<br>FDA 0(6)<br>EUCAST 0(6)	0.06	1	≤0.03 to 1
		A. baumannii complex[b]	Carbapenem-resistant	CLSI 7(14)<br>FDA -(14)<br>EUCAST 64(14)	4	8	0.06
		P. aeruginosa	Carbapenem-resistant	CLSI 0(14)<br>FDA 29(14)<br>EUCAST 29(14)	1	8	0.5 to 8
		S. maltophilia	-	CLSI 0(11)<br>FDA -(11)<br>EUCAST -(11)	0.12	0.25	≤0.03 to 0.5
		Achromobacterxylosidan	-	CLSI -(8)<br>FDA -(8)<br>EUCAST 0(18)	0.25	1	0.06 to 1
		B.cepacia complex	-	CLSI -(3)<br>FDA -(3)<br>EUCAST 0(3)	0.12	0.25	0.12 to 0.25
Gorden[14]<br>[2020]	Canada<br>2015-2017<br>CLSI 2019	*E. coli*	-<br>ESBL<br>AmpC	0(177)<br>0(29)<br>0(6)	≤0.03<br>0.25<br>≤0.03	0.5<br>-<br>-	≤0.03 to 2
		K. pneumoniae	-<br>ESBL	0(121)<br>0(11)	≤0.03<br>1	0.25<br>-	≤0.03 to 4

		E. cloacae	-	0(93)	0.12	1	≤0.03 to 2
		K. oxytoca	-	0(46)	≤0.03	0.12	≤0.03 to 0.5
		K. aerogene	-	0(24)	0.06	-	≤0.03 to 0.5
		Other *Enterobacterales*	Ertapenem-nonsusceptible	0(21)	0.25	-	≤0.03 to 4
		A. baumannii	-	0(11)	0.06	-	≤0.03 to 0.25
		P. aeruginosa	-<br>Meropenem-nonsusceptible<br>MDR	0(201)<br>0(54)<br>0(29)	0.06<br>0.12<br>0.12	0.5<br>1<br>-	≤0.03 to 2<br>≤0.03 to 1<br>≤0.03 to 1
		S. marcescens	-	0(47)	≤0.03	0.25	≤0.03 to 1
		Proteus mirabilis	-	0(14)	≤0.03	-	≤0.03 to 0.25
		S. maltophilia	-	0(66)	0.12	0.5	≤0.03 to 4
Shazad[28] [2020]	United Kingdom 1990s, 2008-2018 EUCAST 2020	*Enterobacterales*	NDM<br>KPC<br>OXA-48<br>VIM<br>ESBL + porin loss<br>AmpC + porin loss<br>IMP<br>GES, IMI, or SME	59.0(61)<br>8.9(56)<br>7.1(56)<br>19.1(47)<br>38.5(26)<br>0(25)<br>6.7(15)<br>0(19)	-	-	0.25 to 32<br>≤0.03 to 8<br>≤0.03 to 8<br>≤0.03 to 8<br>0.125 to 32<br>0.06 to 2<br>≤0.03 to 4<br>0.06 to 2
		A. baumannii[b]	NDM<br>OXA-23<br>OXA-51<br>OXA-58<br>OXA-24/40	50(20)<br>14.6(41)<br>5.3(19)<br>10(10)<br>11.1(9)	-	-	1 to ≥128<br>0.06 to ≥128<br>0.06 to 16<br>0.06 to ≥128<br>0.25 to 4

		P. aeruginosa	NDM<br>VIM<br>IMP<br>GES<br>PER<br>VEB	54.5(11)<br>6.7(30)<br>20(25)<br>10(20)<br>33.3(15)<br>10(10)	-	-	1 to ≥128<br>≤0.03 to ≥128<br>0.06 to 16<br>0.06 to 4<br>0.06 to 16<br>0.05 to 8
Kresken[11]<br>[2020]	Germany<br>2013-2014<br>2014-2017<br>EUCAST 2020	*Enterobacterales*	2010-2017: Carbapenemase-producing	16.7(30)	1	4	≤ 0.03 to 16
		E. coli	2013-2014	2.6(38)	0.06	1	≤ 0.03 to 4
		K. pneumoniae	2013-2014	8(25)	≤ 0.03	2	≤ 0.03 to 8
		E. cloacae complex	2013-2014	9.5(21)	0.25	1	0.12 to 4
		K. oxytoca	2013-2014	0(14)	≤ 0.03	0.5	≤ 0.03 to 1
		Morganellaceae	2013-2014	0(14)	≤ 0.03	0.25	≤ 0.03 to 5
		Citrobacter spp.	2013-2014	0(11)	0.25	0.5	≤ 0.03 to 1
		Other *Enterobacterales*	2013-2014: 2 *Hafnia alvei*, 4 *K. aerogenes*	0(6)	-	-	0.06 to 8
		A. baumannii[b]	2010-2017: OXA-23/-24<br>Carbapenemase-producing	0(7)	-	-	0.06 to 0.25
		A. baumannii group[b]	2013-2014: 7 *A. baumannii*, 6 *A. pittii*	(0)13	0.06	0.12	0.06 to >0.12
		P. aeruginosa	2013-2014<br>2010-2017: Carbapenemase-producing	0(54)<br>9.1(22)	0.12<br>0.5	0.5<br>2	≤ 0.03 to 1<br>0.12 to 8
		S. marcescens	2013-2014	0(17)	0.25	0.5	0.06 to 0.5
Mariana[21]<br>[2020]		*Enterobacterales*	ID-CAMHB<br>AD	5,14,33(335)<br>23,35,47(335)	2	8<br>32	0.015 to >64<br>0.008 to >64

	United States, Canada and Singapore 1996-2013,2015 CLSI 2020 FDA 2019 EUCAST 2020	*E. coli*	ID-CAMHB<br>AD	2,5,17(99)<br>8,16,30(99)	1	4<br>8	0.03 to >64<br>0.008 to >64
		K. pneumoniae	ID-CAMHB<br>AD	3,12,38(166)<br>31,42,51(166)	2	8<br>32	0.03 to >64<br>0.03 to >64
		E. cloacae	ID-CAMHB<br>AD	17,44,53(36)<br>39,50,64(36)	4	16<br>32	0.25 to 32<br>0.06 to 64
		Other *Enterobacterales*	ID-CAMHB<br>AD	6,18,36(34)<br>21,38,53(34)	1<br>4	8<br>32	0.015 to 64<br>0.03 to 64
		A. baumannii	ID-CAMHB<br>AD	33, -, -(97)<br>46, -, -(97)	2<br>8	32<br>64	0.015 to >64<br>0.125 to >64
		P. aeruginosa	ID-CAMHB<br>AD	3,3,3(58)<br>3,30,30(58)	0.5<br>2	2<br>8	0.008 to 32<br>0.06 to 32
		S. maltophilia	ID-CAMHB<br>AD	0, -, -(71)<br>0, -, -(71)	0.06<br>0.25	0.25<br>1	0.004 to 0.5<br>0.015 to 4
		B.cepacia complex	ID-CAMHB<br>AD	-, -, -(49)<br>-, -, -(49)	0.03<br>0.06	1<br>1	0.008 to >64<br>0.015 to >64
Christopher[10] [2020]	Europe 2014-2016 EUCAST 2020	*Enterobacterales*	KPC<br>VIM<br>NDM<br>OXA-48	16.4(238)<br>21.0(62)<br>48.6(37)<br>11.8(85)	1<br>1<br>2<br>1	4<br>4<br>4<br>4	0.03 to 8<br>0.06 to 8<br>1 to 32<br>0.015 to 4
		A. baumannii[b]	OXA-23<br>OXA-24/40<br>OXA-58<br>GES<br>NDM	3.7(135)<br>6.8(88)<br>0(6)<br>0(1)<br>-(3)	0.25<br>0.25<br>-<br>-<br>-	5<br>2<br>-<br>-<br>-	0.015 to 8<br>0.03 to ≥64<br>0.12 to 2<br>0.5<br>2 to 8
		P. aeruginosa	GES<br>VIM<br>NDM<br>Non-carpanemase-producing	0(12)<br>0(73)<br>0(6)<br>3.4(88)	0.12<br>0.12<br>-<br>0.25	0.25<br>0.5<br>-<br>1	0.06 to 0.5<br>0.015 to 2<br>0.25 to 0.5<br>0.04 to 8

James A[16]<br>[2019]	Europe and<br>North America<br>2015-2016	*E. coli*	North America<br>Europe	0(748)<br>0(1082)	0.12	0.5<br>1	≤0.002 to 4<br>≤0.002 to 8
		K. pneumoniae	North America<br>Europe	0(614)<br>0(914)	0.12<br>0.25	0.5<br>2	≤0.002 to 4
		E. cloacae	North America<br>Europe	-(214)<br>0(380)	0.25<br>0.5	1	0.008 to 128<br>≤0.002 to 4
		E. aerogenes	North America<br>Europe	0(114)<br>0(130)	0.12<br>0.06	0.5	≤0.004 to 2<br>≤0.002-4
		K. oxytoca	North America<br>Europe	0(149)<br>0(240)	0.06	0.5	≤0.002 to 2
		Klebsiella variicola	North America	0(35)	0.03	0.25	≤0.002 to 2
		C. koseri	North America<br>Europe	0(56)<br>0(152)	0.5	1<br>0.5	≤0.015 to 8<br>≤0.012 to 1
		C. freundii	North America<br>Europe	0(100)<br>0(152)	0.12	1	≤0.004 to 4<br>≤0.002 to 8
		A. baumannii	North America<br>Europe	-(223)<br>-(614)	0.25	2	≤0.008 to >256<br>≤0.002 to >256
		Acinetobacter pitii	North America<br>Europe	0(66)<br>0(45)	0.12	0.5	≤0.002 to 1<br>0.008 to 1
		P. aeruginosa	North America<br>Europe	0(619)<br>0(921)	0.12	0.5	≤0.002 to 4<br>≤0.002 to 8
		S. maltophilia	North America<br>Europe	-(165)<br>-(175)	0.12	0.5	0.004 to 64<br>≤0.002 to 64
		S. marcescens	North America<br>Europe	-(368)<br>-(408)	0.12	0.5	≤0.015 to 32<br>≤0.06 to >64
		B. cepacia complex	North America<br>Europe	-(40)<br>-(49)	0.015	0.12<br>0.5	≤0.002 to 32

Shun-Chung[17] [2019]	China 2016-2017	*A. baumannii*	Imipenem-resistant	7(100)	0.5	8	0.06 to >64
		P. aeruginosa	Imipenem-resistant<br>Meropenem-susceptible	0(100)<br>0(25)	0.12<br>0.06	2<br>0.5	≤ 0.03 to 8<br>≤ 0.03 to 2
		S. maltophilia	Imipenem-resistant	0(100)	0.06	0.25	≤ 0.03 to 1
Michael R[3] [2018]	Worldwide 2018	*Enterobacteriaceae*	None<br>KPC-2<br>KPC-3<br>KPC-4<br>NDM<br>OXA-48<br>NDM + OXA-48<br>Other	0(18)<br>-(355)<br>-(380)<br>-(2)<br>-(28)<br>0(7)<br>-(1)<br>-(43)	≤0.03<br>1<br>0.25<br>0.5<br>2<br>0.25<br>-<br>2	≤0.03<br>8<br>2<br>16<br>8<br>1<br>-<br>8	≤0.03 to 4<br>≤0.03 to 32<br>≤0.03 to 64<br>0.5 to 16<br>0.25 to >64<br>≤0.03 to 1<br>1<br>≤0.03 to >64
		A. baumannii	Carbapenem-susceptible<br>Carbapenem-resistant	-(99)<br>-(101)	0.12<br>0.25	0.5<br>1	≤0.03 to >64<br>≤0.03 to >64
		P. aeruginosa	Carbapenem-resistant	0(27)	0.25	0.5	≤0.03 to 1
		S. maltophilia	Carbapenem-resistant	0(25)	0.006	0.25	≤0.03 to 0.25
Meredith A[18] [2018]	North America andEurope 2014-2016	*E. coli*	Carbapenem-nonsusceptible	0(73)	1	2	≤0.015 to 4
		K. pneumoniae	Carbapenem-nonsusceptible	-(689)	1	4	0.004 to 32
		Other *Enterobacter spp.*	137 *E. cloacae,* 13 *E. aerogenes,* 5 *Enterobactekobei and* 3 *Enterobacter asburiae,* Carbapenem-nonsusceptible	-(158)	2	8	0.06 to 32
		K. oxytoca	Carbapenem-nonsusceptible	0(31)	0.25	1	0.03 to 4
		S. marcescens	Carbapenem-nonsusceptible	0(39)	0.5	2	0.015 to 4

		Citrobacter spp.	Carbapenem-nonsusceptible	0(32)	0.5	2	0.015 to 8
		A. baumannii	MDR	-(368)	0.25	8	0.015 to >256
		P. aeruginosa	MDR, ceftolozane-tazobactam-nonsusceptible<br>MDR, ceftazidime-avibactam-nonsusceptible	-(199)<br>-(167)	0.25	2	0.015to32<br>0.015 to 32
		S. maltophilia	-	0(217)	0.06	0.25	0.004 to 2
		B. cepacia	-	0(4)	-	-	0.004 to 8
Krystyna M[30]<br>[2018]	North America and Europe<br>2014-2015	*Enterobacteriaceae*	KPC, meropenem-resistant<br>GES,<br>meropenem-resistant+carbapenemase-positive<br>VIM, meropenem-resistant<br>NDM, meropenem-resistant<br>OXA-48, meropenem-resistant<br>Non-carbapenemase, meropenem-resistant	0(75)<br>0(1)<br>0(27)<br>0(12)<br>0(32)<br>0(13)	1<br>-<br>1<br>4<br>0.5<br>0.12	2<br>-<br>4<br>8<br>4<br>2	0.03 to 4<br>0.25<br>0.12 to 4<br>1 to 8<br>0.03 to 4<br>0.08 to 4
		A. baumannii	GES, ESBLs, meropenem-resistant<br>NDM, meropenem-resistant<br>OXA-23, meropenem-resistant<br>OXA-24, meropenem-resistant<br>OXA-58, meropenem-resistant<br>Non-carbapenemase+meropenem-resistant	0(7)<br>0(2)<br>0.37(543)<br>2.4(124)<br>0(14)<br>0(86)	-<br>-<br>0.12<br>0.12<br>0.06<br>0.025	-<br>-<br>1<br>1<br>1<br>2	0.25 to 8<br>1 to 1<br>≤0.002 to 16<br>0.004 to 64<br>0.06 to 1<br>0.008 to 8
		P. aeruginosa	GES, meropenem-resistant<br>IMP, meropenem-resistant<br>VIM, meropenem-resistant<br>Non-carbapenemas+meropenem-nonsusceptible	0(4)<br>0(4)<br>0(26)<br>0(319)	-<br>-<br>0.25<br>0.12	-<br>-<br>2<br>0.5	0.12 to 0.25<br>1 to 2<br>0.008 to 2<br>≤0.002 to 4
Dobias[27]<br>[2017]	Worldwide<br>2000-2016	*E. coli*	KPC<br>OXA-48<br>NDM, VIM or IMP	-(12)<br>-(42)<br>-(67)	0.5<br>0.06<br>1	1<br>0.5<br>16	0.03 to 64

		K. pneumoniae	KPC<br>OXA-48<br>NDM, VIM or IMP	-(101)<br>-(88)<br>-(38)	1<br>0.25<br>1	2<br>1<br>4	0.03 to 64
		Other *Enterobacter sp.*	KPC<br>OXA-48<br>NDM, VIM or IMP	-(14)<br>-(24)<br>-(29)	0.5<br>1<br>1	1<br>4<br>4	0.03 to 64
		A. baumannii	OXA	-(85)	0.12	4	0.03 to 64
		P. aeruginosa	IMP/KPC/VIM/ SPM or GIM	-(30)	0.5	2	0.03 to 64
Meredith A[19] [2017]	North America and Europe 2014-2015	*Enterobacteriaceae*	-<br>Meropenem-nonsusceptible	**0(3080)**<br>0(139)	0.12<br>1	14	≤0.002 to 8<br>≤0.008 to 8
		E. coli	North America<br>Europe	0(740)<br>0(789)	0.06<br>0.12	0.25<br>0.5	≤0.002 to 2<br>≤0.002 to 4
		Klebsiella spp.	North America<br>Europe	0(1010)<br>0(1021)	0.06<br>0.12	0.25<br>2	≤0.002 to 4<br>≤0.002 to 8
		Enterobacter spp.	North America<br>Europe	0(494)<br>0(530)	0.25	1	0.004 to 4<br>≤0.008 to 8
		Serratia spp.	North America<br>Europe	0(503)<br>0(493)	0.06<br>0.12	0.25<br>0.5	≤0.002 to 8<br>≤0.002 to 8
		Citrobacter spp.	North America<br>Europe	0(260)<br>0(247)	0.12	0.25<br>0.5	≤0.002 to 2<br>0.004 to 4
		P. aeruginosa	North America<br>North America: Meropenem-nonsusceptible<br>Europe<br>Europe: Meropenem-nonsusceptible	**0(765)**<br>0(151)<br>**0(765)**<br>0(202)	0.06<br>0.06<br>0.12<br>0.25	0.5<br>0.5<br>0.5<br>1	≤0.002 to 8<br>≤0.002 to 4<br>≤0.002 to 4<br>≤0.008 to 4

		A. baumannii	North America<br>North America: Meropenem-nonsusceptible<br>Europe<br>Europe: Meropenem-nonsusceptible	**0(309)**<br>-(173)<br>**-(839)**<br>-(595)	0.12<br>0.12<br>0.25<br>0.12	1	≤0.002 to 8<br>≤0.002 to 64<br>≤0.002 to<br>8≤0.004 to 64
		S. maltophilia	North America<br>Europe	0(152)<br>0(276)	0.06	0.5<br>0.25	≤0.002 to 4<br>0.04 to 2
		B. cepacia	North America<br>Europe	-(6)<br>0(6)	-	-	0.015 to 16<br>0.004 to 1
Naoki[29]<br>[2016]	Worldwide<br>2000-2009	*E. coli*	NDM-1<br>KPC<br>ESBL<br>Other	26.3(19)<br>0(7)<br>0(49)<br>0(3)	-	-	0.25 to >16<br>≤0.125 to 4<br>≤0.125 to 1<br>≤0.125
		K. pneumoniae	NDM-1<br>KPC<br>ESBL<br>VIM-1<br>Other	0(24)<br>0(20)<br>0(24)<br>0(4)<br>0(9)	-	-	≤0.125 to 2<br>≤0.125 to 1<br>≤0.125 to 2<br>≤0.125<br>≤0.125 to 0.5
		S. marcescens<br>*C. freundii*<br>*E. cloacae*	NDM-1<br>KPC<br>VIM<br>IMP<br>ESBL<br>Other	0(6)<br>0(20)<br>0(8)<br>0(8)<br>0(19)<br>0(13)	-	-	≤0.125 to 2<br>≤0.125 to 4<br>≤0.125 to 16<br>≤0.125 to 16<br>≤0.125 to 4<br>≤0.125 to 2
	Worldwide<br>2009-2011	*E. coli*	-	0(106)	0.125	1	≤0.063 to 4
		K. pneumoniae	-	0(105)	≤0.063	0.125	≤0.063 to 2
		E. cloacae	-	0(100)	0.125	1	≤0.063 to 16
		E. aerogenes	-	0(100)	≤0.063	0.5	≤0.063 to 8

		C. freundii	-	-(100)	≤0.063	0.125	≤0.063 to >64
		S. marcescens	-	-(103)	≤0.063	≤0.063	≤0.063 to >64
Akinobu[32] [2015]	Worldwide 2000-2009	*A. baumannii*	Carbapenem-resistant<br>IMP-1<br>OXA-23<br>OXA-24<br>OXA-51/ISAba1<br>OXA-58	**-(29)**<br>0(2)<br>16.7(12)<br>0(8)<br>0(2)<br>0(5)	0.5<br>-<br>-<br>-<br>-<br>-	8<br>-<br>-<br>-<br>-<br>-	0.03 to >32<br>0.12 to 0.25<br>0.03 to >32<br>0.12 to 8<br>0.5 to 1<br>0.12 to 4
		P. aeruginosa	MBL producing<br>GIM-1<br>IMP<br>SPM-1<br>VIM	**0(33)**<br>0(1)<br>0(13)<br>0(3)<br>0(16)	0.5<br>-<br>-<br>-<br>-	4<br>-<br>-<br>-<br>-	0.03 to 8<br>0.5<br>0.12 to 1<br>0.03 to 2<br>0.06 to 8
	Worldwide 2009-2011	*A. baumannii*	-	0(104)	0.125	2	≤0.063 to 4
		P. aeruginosa	-	0(104)	≤0.063	1	≤0.063 to 4
		S. maltophilia	-	0(108)	0.125	0.5	≤0.063 to 4

[a]CLIS 2020 was used to determine the resistance rate when the criteria were not provided by the original study.
[b]Non-species special (PK-PD) breakpoints.
